# The Complex Interplay of TGF-β and Notch Signaling in the Pathogenesis of Fibrosis

**DOI:** 10.3390/ijms251910803

**Published:** 2024-10-08

**Authors:** Nadezhda Bakalenko, Evdokiya Kuznetsova, Anna Malashicheva

**Affiliations:** Institute of Cytology, Russian Academy of Sciences, St-Petersburg 194064, Russia; bakalenko@gmail.com (N.B.); dunfrogg@gmail.com (E.K.)

**Keywords:** fibrosis, Notch signaling, TGF-β pathway, signaling crosstalk, SMAD, NICD

## Abstract

Fibrosis is a major medical challenge, as it leads to irreversible tissue remodeling and organ dysfunction. Its progression contributes significantly to morbidity and mortality worldwide, with limited therapeutic options available. Extensive research on the molecular mechanisms of fibrosis has revealed numerous factors and signaling pathways involved. However, the interactions between these pathways remain unclear. A comprehensive understanding of the entire signaling network that drives fibrosis is still missing. The TGF-β and Notch signaling pathways play a key role in fibrogenesis, and this review focuses on their functional interplay and molecular mechanisms. Studies have shown synergy between TGF-β and Notch cascades in fibrosis, but antagonistic interactions can also occur, especially in cardiac fibrosis. The molecular mechanisms of these interactions vary depending on the cell context. Understanding these complex and context-dependent interactions is crucial for developing effective strategies for treating fibrosis.

## 1. Fibrosis Development

### 1.1. Cellular and Molecular Mechanisms

Fibrosis is a pathological condition characterized by the excessive growth of connective tissue and the abnormal deposition of extracellular matrix (ECM). Fibrotic tissue replacement leads to the gradual loss of specific tissue properties and causes the dysfunction of the affected organ [1,2]. Fibrosis can occur in nearly any organ including the liver, kidney, lung, heart, and skin [3,4,5,6,7]. Fibrosis can be induced by a variety of conditions, including past illnesses, traumas, surgeries, radiation, and allergic reactions [8,9,10,11,12]. The most common reason for fibrogenesis is excessive, prolonged, or recurrent tissue injury accompanied by chronic inflammation [13,14,15].

**Figure 1:**
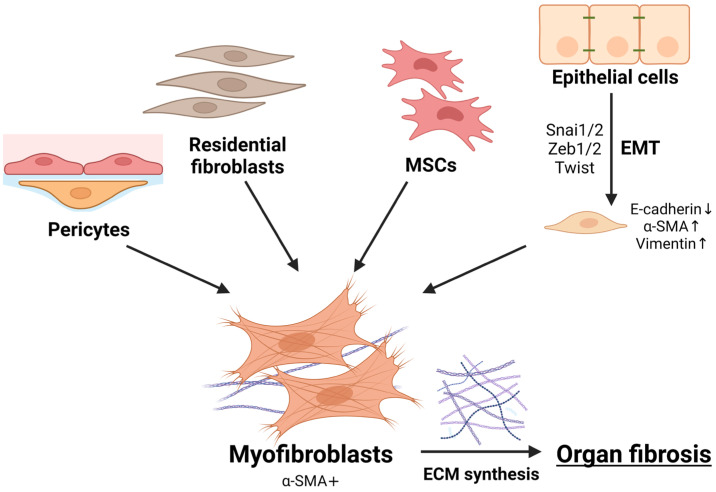
Cellular sources of myofibroblasts and fibrosis development. MSCs—mesenchymal stem cells; EMT—epithelia–mesenchymal transition; ECM—extracellular matrix. “Created with BioRender.com”.

Many cell types are involved in the complex, multicomponent mechanisms of fibrogenesis, but myofibroblasts play a central role. Myofibroblasts are characterized by a high level of α-smooth muscle actin (α-SMA) expression. Myofibroblasts synthesize large amounts of ECM, such as collagens and fibronectin, and produce fibrogenic cytokines [16,17]. The cellular sources of myofibroblasts accumulation in fibrosis are various. They can originate from residential fibroblasts and pericytes, from mesenchymal stem cells in the bone marrow, and via the transdifferentiation of epithelial cells [18,19,20]. The last mechanism involves epithelia–mesenchymal transition (EMT), which results in the transformation of epithelial cells into mesenchymal cells (Figure 1). At the molecular level, this process is characterized by the expression of the transcription factors Snai1 and Snai2, Twist, Zeb1, and Zeb2. This leads to a downregulation of E-cadherin, involved in the formation of tight junctions between epithelial cells, and to an upregulation of mesenchymal markers such as α-SMA and vimentin. The cells become motile and acquire a mesenchymal phenotype [21,22]. Numerous studies have demonstrated that EMT is an essential component in the development of fibrosis [13,23,24,25].

Extensive studies on the molecular mechanisms underlying fibrosis have shown that numerous factors and signaling pathways involved in organ development, such as Notch, Wnt, TGF-β, etc., also participate in fibrogenesis [26,27,28]. The interplay between all these pathways remains unclear, and we are still far from a complete understanding of the full signaling network underlying fibrosis. This review discusses the crosstalk between two important signaling pathways, TGF-β and Notch, in the pathogenesis of fibrosis.

### 1.2. TGF-β Pathway and Its Role in Fibrosis

**Figure 2:**
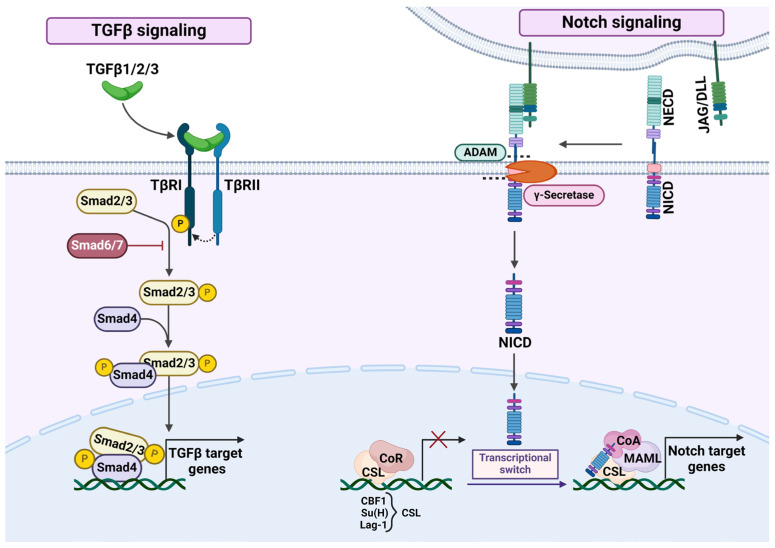
TGF-β and Notch signaling pathways. TGF-β—transforming growth factor β; TβRI—TGF-β receptor 1; TβRII—TGF-β receptor 2; NECD—Notch extracellular domain; NICD—Notch intracellular domain; DLL—distal-less like; JAG—Jagged; DLL and JAG are Notch ligands; ADAM—ADAM metallopeptidase; ADAM and γ-secretase are needed to cleave Notch receptor and release NICD; CSL—NICD co-factor, its name is an abbreviation of the first letters of the members of its family from different animal groups: CBF-1/RBPJ (mammalian), Su(H) (drosophila), Lag-1 (nematoda); CoR—co-repressor; CoA—co-activator. See text for detail. “Created with BioRender.com”.

TGF-β signaling is facilitated by transmembrane serine/threonine kinase receptors, specifically type II (TβRII) and type I (TβRI) receptors. The TGF-β ligand family includes TGF-β1, TGF-β2, and TGF-β3. TGF-β ligands bind to TβRII, which recruits and phosphorylates TβRI. The TβRI then phosphorylates receptor-regulated SMADs (SMAD1, SMAD2, or SMAD3). They are also known as R-SMADs, which can now form a complex with coSMAD (SMAD4), which translocates into the nucleus. There, they interact with other transcription factors to either activate or repress the transcription of TGF-β target genes. Inhibitory SMADs (I-SMADs), SMAD6 and SMAD7, act by binding to TβRI, thereby preventing the recruitment and phosphorylation of the R-SMADs [29,30] (Figure 2).

The TGF-β signaling pathway plays an important role in the development, homeostasis, and repair of most body tissues. It is involved in controlling proliferation, differentiation, migration, and apoptosis of many cell types [31,32,33].

All three isoforms of TGF-β, TGF-β1, TGF-β2, and TGF-β3 have fibrogenic effects on different cell types [26]. Among them, TGF-β1 is considered to play a major role in fibrogenesis and mediate part of the functions of TGF-β2 and TGF-β3 [34]. TGF-β1 can induce EMT, myofibroblast differentiation, and fibrosis of any tissue or organ [35]. α-SMA contains SMAD3 binding element in its promoter, which is required for transcriptional activation of α-SMA by TGF-β1 [36]. R-SMAD complexes can directly bind to the Snai1 promoter to initiate its transcription and can also form complexes with Snai1 protein to repress the expression of E-cadherin and occludin [37]. TGF-β1 can also directly induce collagen 1 alpha 1 (COL1A1) transcription [1,38,39].

Beyond the SMAD-dependent pathway there are various non-SMAD downstream signaling pathways for TGF-β1 ligands. Some of them, like the mitogen-activated protein kinase (MAPK) pathway, extracellular signal-regulated kinase (ERK)1/2 pathway, and c-Jun N-terminal kinase (JNK) pathway, are involved in TGF-β1-induced renal [40] and lung [41] fibrosis. It is widely recognized that the TGF-β signaling pathway plays a central and crucial role in fibrogenesis of any organ or tissue [1,26,29,42].

### 1.3. Notch Signaling and Fibrogenesis

The Notch signaling pathway plays a crucial role in determining cell fate and is activated through direct cell-to-cell contact. The Notch receptor on one cell binds to a transmembrane ligand, such as Jagged or Delta-like, present on a neighboring cell. This interaction initiates the cleavage that releases Notch intracellular domain (NICD). The NICD then moves into the nucleus where it forms a complex with CSL protein (CBF-1/RBPJ, Su(H), Lag-1). CSL is a transcription regulator, which acts as a repressor when not bound to NICD and as an activator when bound to NICD. NICD-CSL complex promotes the expression of Notch target genes, firstly members of Hes, and Hey gene families, which encode basic helix-loop-helix (bHLH) transcription factors essential for mediating Notch’s downstream effects (Figure 2). Mammals have four Notch receptors (Notch1-4), with their intracellular domains (N1ICD, N2ICD, N3ICD, and N4ICD), five ligands—two Jagged family ligands (JAG1 and JAG2), and three delta-like ligands (DLL1, DLL3, and DLL4) [43,44,45].

Over the last two decades, evidence has accumulated on the involvement of Notch signaling in the fibrosis of various organs and tissues. Like the TGF-β pathway, Notch signaling is capable of regulating EMT and myofibroblast activation in lung, kidney, liver, skin, and other organs [13,46,47,48,49]. For example, all four Notch receptors can initiate fibroblast to myofibroblast transition in primary human alveolar fibroblast cultures [50]; Notch1, Notch3, and Jag-1 are involved in renal, liver, and skin fibrosis [13,51,52,53]. However, the role of Notch signaling in fibrogenesis is not so straightforward. The Notch signaling pathway demonstrates cardioprotective effects after myocardial infarction (MI), in particular, it can attenuate the profibrotic changes [54,55,56,57,58]. On the other hand, some studies suggest that Notch signaling has the ability to induce cardiac fibrosis. In transgenic mice subjected to myocardial infarction with increased afterload, activation of Notch through the immobilized DLL4 ligand promotes the differentiation of multipotent stromal cells into a myofibroblastic phenotype, originating from the epicardial cell population [59]. Notch signaling activation induces the expression of Snai2 and α-SMA in cardiac mesenchymal cells [60]. Therefore, Notch signaling plays a complex role in fibrosis, contributing to fibrogenesis in various organs but also demonstrating cardioprotective effects by attenuating fibrosis after MI.

## 2. Notch and TGF-β Signaling Pathways Interplay in the Fibrosis of Various Organs

### 2.1. Notch Signaling and TGF-β Pathway Crosstalk in Pulmonary Fibrosis

There is substantial evidence that both Notch and TGF-β pathways play significant roles in the development of lung fibrosis [23,61,62,63]. Several studies suggest an interaction between these signaling pathways in the induction of pulmonary fibrosis.

For instance, Notch1 induces the production of TGF-β1 and the phosphorylation of SMAD3 that activate the expression of α-SMA in rat alveolar epithelial cell line RLE-6TN. In turn, the addition of TGF-β increases the expression level of Notch1. Inhibition of the Notch signaling after TGF-β1 treatment significantly reduces the effect of TGF-β1 on α-SMA activation, and α-SMA induction by N1ICD is completely blocked by an inhibitor of SMAD2/3 phosphorylation. Thus, in RLE-6TN cells, Notch and TGF-β signaling act synergistically during myofibroblast differentiation. Activation of one pathway triggers the other, and both are essential for the upregulation of α-SMA [18]. The interaction with TGF-β has been shown for another Notch receptor—Notch3 in mouse and human lung fibroblasts. Primary lung fibroblasts isolated from Notch3 knockout (Notch3-KO) mice exhibit a weak response to TGF-β1 stimulation. The number of α-SMA-positive cells was markedly decreased in Notch3-KO cells at multiple time points, specifically 24 h, 48 h, 72 h, and 5 days after treatment with TGF-β1, indicating reduced myofibroblast differentiation [64]. Incubation with TGF-β1 leads to an increase in the expression of Notch3 in human lung fibroblasts IMR-90 [41]. In alveolar epithelial cells from the human adenocarcinoma cell line A549, activation of the TGF-β pathway also leads to an increased expression of Notch2, Notch4, and the ligand Jagged1. Inhibition of Notch signaling significantly diminishes TGF-β1-induced EMT by suppressing the activation of Snai1. Additionally, E-cadherin expression remains significantly higher in cells with depleted Notch2, Notch4, or Jagged1, and this effect is only observed in the presence of TGF-β1 [65]. Moreover, overexpression of the intracellular domain of Notch4 (N4ICD) in primary cultures of human fibroblasts increases expression of TGF-β1 and phosphorylation of SMAD2 [66]. Studies in mouse models of experimentally induced lung fibrosis have demonstrated that the Jagged1/Notch and TGF-β1/SMAD pathways work together to promote EMT and myofibroblast differentiation [16,67,68]. Together, these studies highlight the cooperative and synergistic effects of the TGF-β and Notch pathways in lung fibrogenesis [62].

### 2.2. Notch and TGF-β in Liver Fibrosis

Fibrosis is a key pathological process in the development of all chronic liver diseases [69,70]. A key aspect of liver fibrogenesis is the activation of hepatic stellate cells (HSCs), which plays a pivotal role in this process. TGF-β1 treatment of mouse HSCs promotes their activation and differentiation into myofibroblasts [71,72] and this transformation is accompanied by increased expression of Notch1, Jagged1, and Hes1 [73]. Inhibition of Notch signaling hinders myofibroblast differentiation induced by TGF-β1, suggesting that TGF-β1 signaling controls HSCs activation through regulating the expression of the Notch pathway [73]. Further evidence indicates that Notch signaling can operate upstream of TGF-β1. For example, overexpression of micro-RNA-25 (miR-25), which targets key components of Notch signaling such as ADAM-17 (ADAM Metallopeptidase Domain 17) and FKBP14 (FKBP Prolyl Isomerase 14), leads to a reduction in the expression of TGF-β1 and TGFβR1 in the HSC cell line LX-2. Notably, in inactive LX-2 cells, miR-25 expression does not affect the levels of activation markers α-SMA and Col1A1. However, when LX-2 cells are stimulated with TGF-β1, overexpression of miR-25 significantly inhibits the upregulation of Col1A1, while it has no effect on the increased expression of α-SMA [74]. In vivo studies also support the interaction between these pathways. Rats with concanavalin A-induced liver fibrosis exhibit higher expression levels of Notch (Notch1, Hes1, Hes5) and TGF-β (TGF-β1, SMAD3) pathway components than control rats. Inhibition of Notch signaling in these cells leads to the downregulation of TGF-β1 and SMAD3, while TGF-β inhibitors, in turn, suppress Notch1, Hes1, and Hes5 [75]. Additionally, the suppression of Notch signaling results in a notable decrease in fibrogenesis within the intrahepatic cholangiocarcinoma microenvironment, especially in cancer-associated fibroblasts (CAFs). The deactivation of CAFs and reduction of ECM synthesis in the presence of a Notch inhibitor are mediated through the inhibition of canonical TGF-β signaling [76]. Taken together, these findings highlight the positive cross-regulation between the Notch and TGF-β signaling pathways in liver fibrosis, underscoring their synergistic role in promoting the fibrotic process.

### 2.3. Notch and TGF-β Interaction in Kidney Fibrosis

The interplay between Notch and TGF-β is essential for renal fibrosis [53,77]. The profibrotic effect of these pathways were demonstrated in both interstitial fibroblasts and tubular epithelial cells (TECs). Renal interstitial fibroblasts are capable of fibroblast-to-myofibroblast transformation, leading to fibrosis development [78]. TECs secrete pro-fibrotic cytokines. In vitro TECs can undergo EMT, but there are no solid data supporting EMT as an in vivo process in kidney fibrosis [79]. The impact of Notch signaling on TGF-β has been demonstrated both in vivo and in vitro using the unilateral ureteral obstruction (UUO) mouse model. The UUO mice exhibited increased expression of Notch1, Notch3, and Notch4, and its target genes Hes1 and HeyL. Inhibition of Notch signaling with dibenzazepine (DBZ) significantly reduced fibrotic tissue transformation and the expression of fibrogenesis markers, such as collagens, α-SMA, and fibronectin, and it also suppressed TGF-β1 expression and SMAD2 and SMAD3 phosphorylation [78]. Tubular epithelial cells (TECs), transfected with a vector, bearing N1ICD, demonstrated enhanced expression of TGF-β1. Conditioned medium from N1ICD-transduced TECs stimulated the renal fibroblasts to express collagens and fibronectin and led to their differentiation to myofibroblasts. The effects of N1ICD were significantly attenuated by adding anti-TGF-β1 neutralizing antibodies to the medium. Together, these findings indicate that Notch activation in TECs drives myofibroblast differentiation by increasing TGF-β1 production [78]. Similar results were obtained on cell cultures from rat and human kidney fibrotic tissues: Notch1 activation in TECs and interstitial fibroblasts contributes to the myofibroblastic phenotype and fibrosis by targeting downstream TGF-β1/SMAD2/3 signaling [80]. Moreover, this study demonstrated that TGF-β1 upregulates Notch1, Jagged-1, and Hey1 expression in TECs and interstitial renal fibroblasts. Numerous studies revealed cooperative interaction of Notch and TGF-β1 pathways in renal fibrosis [81,82]; however, some evidence indicates the possibility of antagonistic relationships of these signals in the renal epithelium. Under conditions of pathological shear stress, which triggers fibrosis in renal proximal tubular epithelial cells (PTECs), Notch4 inhibits the TGF-β1 pathway [83]. Therefore, we can conclude that the interplay between Notch and TGF-β signaling is crucial in kidney fibrosis, where these pathways cooperatively promote EMT and myofibroblast differentiation, but under certain conditions, Notch4 may antagonize TGF-β1 signaling in renal epithelial cells.

### 2.4. Crosstalk between Notch and TGF-β in Cardiac Fibrosis

Cardiac fibrosis is a common pathophysiologic process in most heart disease. Most studies suggests that Notch signaling in cardiac fibroblasts (CFs) and cardiomyocytes suppresses fibrogenesis [55,56,57,58]. The inhibition of Notch signaling with specific antagonist DAPT (γ-secretase inhibitor prevents the release of NICD) in rat CFs results in the fibroblast-to-myofibroblast transformation. Treatment of these cells with TGF-β1 leads to an increase in α-SMA expression and a decrease in the expression of Notch1, Notch3, and Notch4 [56]. Further studies have demonstrated that overexpression of N1ICD reduced all of the TGF-β1-induced profibrotic changes in rat CFs, such as increased proliferation, invasiveness, adhesion, elevated α-SMA expression, and collagen 1 synthesis. Knockdown of the N1ICD, on the contrary, amplified TGF-β1 effects [57]. Overexpression of the Notch ligand Jag-1 in mouse cardiomyocytes resulted in reduced activation of TGF-β2, TGF-β3, and myofibroblast markers after transaortic constriction [55]. The transduction of mouse CFs with lentiviral constructs carrying Notch3 cDNA attenuated TGF-β1-induced fibrosis, while the intramyocardial injection of short interfering RNA for Notch3 (siNotch3), on the contrary, enhanced the profibrotic effects of TGF-β1 [84]. Thus, numerous studies indicated a cardioprotective role of Notch signaling in heart fibrosis, associated with its negative regulation of TGF-β pathway. Nevertheless, some studies have found a synergistic interaction between Notch and TGF-β pathways in cardiac fibrosis and postulated profibrotic effect of Notch signaling [59,85]. Li and colleagues showed using an in vivo MI mouse model that knockdown of the NICD key cofactor CSL using shCSL significantly reduced fibrosis. Mice injected with shCSL exhibited markedly lower levels of TGF-β1 and collagen expression compared to the control group [85]. The interaction between Notch and TGF-β signaling plays a complex role in cardiac fibrosis, with most studies indicating that Notch signaling suppresses TGF-β-induced fibrotic changes, though some evidence suggests a potential synergistic profibrotic effect in certain contexts.

**Figure 3:**
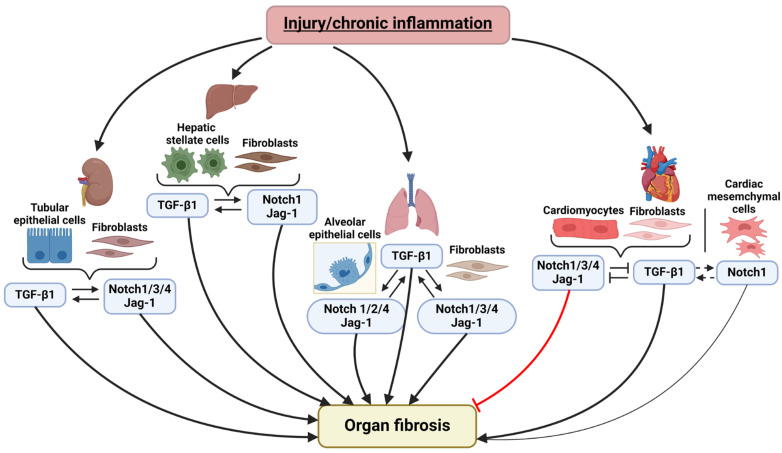
Brief summary of the interactions between TGF-β and Notch signaling pathways in the fibrosis of various organs. TGF-β and Notch act synergistically in the kidney, liver, and lung, leading to myofibroblast differentiation and fibrosis development. The TGF-β pathway induces fibrosis, but the role of Notch signaling in the process of cardiac fibrosis remains ambiguous and highly depends on cell context. Most studies demonstrate that Notch inhibits myofibroblast differentiation via antagonizing TGF-β pathway, but several studies point to possible synergistic interplay between these pathways and demonstrate profibrotic effect of Notch. Jag-1—Jagged-1. “Created with BioRender.com”.

Numerous studies on fibrogenesis across various organs have shown that the TGF-β and Notch signaling pathways generally exhibit functional synergy in the development of fibrosis, as seen in lung, liver, and kidney fibrosis. However, in cardiac fibrosis, Notch signaling displays a dual role: it can act as an antagonist to the TGF-β pathway, preventing the transformation of cardiomyocytes and cardiac fibroblasts into myofibroblasts, or as a protagonist, promoting the differentiation of cardiac mesenchymal cells into a myofibroblastic phenotype (Figure 3).

## 3. Molecular Mechanisms of the Interplay between TGF-β and Notch Signaling

There is limited evidence supporting the direct transcriptional regulation of Notch receptors or ligands by TGF-β-induced SMAD complexes. For example, SMAD2/3 binding to the promoters of Notch2 and Notch3 has been demonstrated in hepatic stellate cells (HSCs) during liver fibrogenesis [86].

Crosstalk of Notch and TGF-β signaling can occur at multiple levels. Below we describe some of the interaction mechanisms, focusing on those involved in fibrogenesis.

### 3.1. Interaction of SMAD3 and NICD

**Figure 4:**
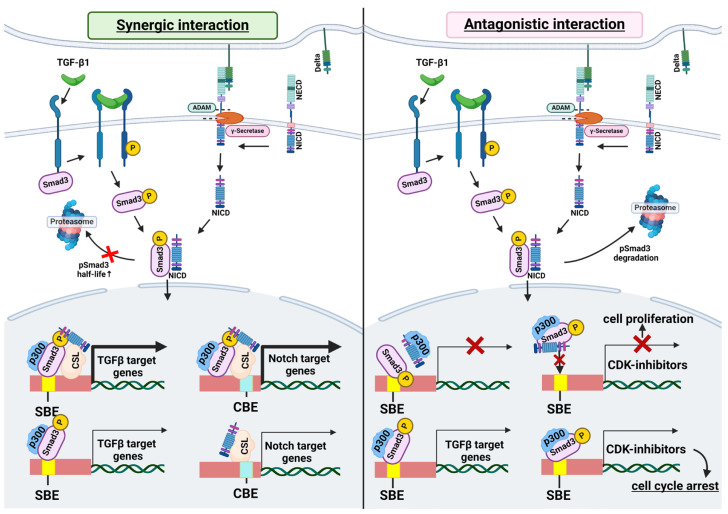
The complex role of NICD-SMAD3 binding in TGF-β and Notch signaling transduction. Synergistic interaction: NICD-pSMAD3 complex moves to the nucleus, interacts with CSL, and enhances transcription of both TGF-β and Notch targets. NICD binding to pSMAD3 can increase the pSMAD3 half-life. Antagonistic interaction: NICD binds to pSMAD3 and sequestrates histone acetyltransferase P300 from it, blocking the transcription of TGF-β transcriptional targets. NICD binding to pSMAD3 can prevent its interaction with target gene promoters. NICD binding to pSMAD3 can promote degradation of the latter. CDK-cyclin-dependent kinase; NECD—Notch extracellular domain; NICD—Notch intracellular domain; SBE—SMAD binding element; CBE—CSL binding element. “Created with BioRender.com”.

The profibrotic effects of TGF-β are primarily mediated through the activation of SMAD3. There are several lines of evidence that NICD can directly bind SMAD3 [57,87,88,89,90]. In 2003 Blokzijl and colleagues showed that N1ICD and phosphorylated SMAD3 (pSMAD3) interact directly in a ligand-dependent manner, and pSMAD3 could be recruited to CSL-binding sites on DNA in the presence of CSL and N1ICD and this complex induces Hes1 expression [87]. A similar cooperation was shown in mouse regulatory T cells in that the N1ICD interacts with activated SMAD3, facilitates its nuclear translocation, where they together upregulate the transcription factor Forkhead box P3 (Foxp3) [91]. The SMAD3-N1ICD-CSL complex can bind to both SMAD and CSL binding sites, leading to histone H4 acetylation on a subset of gene promoters [88]. N1ICD can also prolong the pSMAD3 half-life [88] (Figure 4).

NICD and SMAD3 interaction can also inhibit TGF-β/SMAD3 signal transduction. Indeed, Sun and colleagues demonstrated that direct binding of N4ICD with active SMAD3 reduced the expression of TGF-β/SMAD3 transcriptional targets and attenuated TGF-β1 induced growth inhibition in HC-11 and EpH4 mammary epithelial cells [89]. N1ICD can also inhibit antiproliferative activity of TGF-β in epithelial cells via sequestration of p300 from pSMAD3 [92]. In muscle stem cells N1ICD and pSMAD3 physically interact, and this association prevents pSMAD3 binding to promoters of the cyclin-dependent kinase (CDK) inhibitors p15, p16, p21, and p27, which maintains the proliferative activity of these cells. In aging muscle, N1ICD levels are low, resulting in SMAD3 persistently activating transcription of CDK inhibitors. This leads to the suppression of muscle stem cell proliferation and a restricted capacity for aged muscle regeneration [90]. N1ICD binding with pSMAD3 is capable of preventing SMAD3 interaction with α-SMA promoter region in rat CFs [57]. Some studies suggest that N1ICD and N4ICD can negatively regulate SMAD3 phosphorylation or pSMAD3 degradation [57,83] (Figure 4).

### 3.2. Shared Transcriptional Targets

The genes of the Hey/Hes family of bHLH transcriptional repressors are direct targets of Notch signaling pathway. Sequence analysis of the Hey1 promoter identified five putative consensus binding elements for SMAD3/SMAD4—SMAD-binding element core repeats (SCRs). Zavadil and colleagues showed that TGF-β induced expression of the Hey1 in established cell culture models of TGF-β-induced EMT. TGF-β activated Hey1 via direct binding of the SMAD3/4 complex with its promoter and this activation is Notch independent. The authors made a series of Hey1 promoter deletion constructs and confirmed that promoter fragments containing both proximal and distal SCRs were required for the activation by TGF-β signals, while constructs lacking the SCRs were unresponsive [68]. This work also revealed that TGF-β/SMAD and Notch signals used physically distinct promoter regions to activate Hey1.

It was shown that Notch also can activate classical targets of TGF-β/SMAD pathway, for example, Snai1 and Snai2, which are key players in TGF-β1-induced EMT. Promoter region of Snai1 has one putative CSL binding sequence [65,93]. Constitutive activation of Notch2 or Notch4 was sufficient for the induction of Snai1 in A549 cells. CHiP assay revealed that both N2ICD and N4ICD are recruited to the Snai1 promoter. Interestingly, in human ovarian carcinoma SKOV-3 cells this region was targeted by N1ICD [93]. Notch signaling also directly modulated Snai2 expression that was required for endothelial-to-mesenchymal transition (endoMT) in cardiac cushion morphogenesis [94].

### 3.3. Interactions Mediated by Reactive Oxygen Species (ROS)

TGF-β1 increases ROS production [95], which is important for EMT and fibrosis development [96]. In turn, ROS triggers the expression of some antioxidant factors, including Nrf2 [97]. Several studies have demonstrated that Nrf2 can activate Notch signaling. For instance, Yazaki and colleagues showed that TGF-β1 activates Nrf2 expression in A549 cells in an ROS-dependent manner, and Nrf2 directly activates Notch4 expression via binding with one of the antioxidant response elements (ARE) regions in its promoter [98]. In hepatocytes, activated Nrf2 directly interacts with ARE in the Notch1 promoter to induce its transcription [99]. It is interesting that in A549 cells Notch1 was not induced by TGF-β1 despite the presence of functional ARE [65]. This result suggests the ROS-Nrf2 pathway is necessary but not sufficient for TGF-β1-induced Notch transcription.

In pulmonary fibroblast cell culture IMR-90 TGF-β1-induced activation of Notch3 was also mediated by ROS. The ROS production induced the expression of MAPK kinases p38 and JNK1/2, which in turn upregulated Notch3 receptor expression [41].

## 4. Conclusions

Fibrogenesis of any organ or tissue involves interplay between Notch and TGF-β signaling pathways. The outcomes and mechanisms of this crosstalk vary depending on the cellular context and potentially on the activity of other signaling, such as Wnt and Hippo pathways, which are also involved in regulating similar physiological and pathological processes [100,101,102]. In most cases, TGF-β and Notch signaling pathways demonstrate functional synergy in the development of fibrosis, such as in lung, liver, or kidney fibrosis. However, in cardiac fibrosis, Notch signaling acts as an antagonist to TGF-β pathway, preventing the myofibroblast transformation of heart cells. The diversity of potential interaction mechanisms leads to ambiguous outcomes in the activation of these signaling pathways, complicating the search for therapeutic approaches to treat fibrosis. The development of cell signaling research tools combined with mathematical modeling may reveal a full signaling network in cells under various biological contexts, that will help us accurately predict biological outcomes from combinatorial signaling activities.

## Data Availability

Not applicable.
